# High-Order Interference Effect Introduced by Polarization Mode Coupling in Polarization—Maintaining Fiber and Its Identification

**DOI:** 10.3390/s16030419

**Published:** 2016-03-22

**Authors:** Chuang Li, Jun Yang, Zhangjun Yu, Yonggui Yuan, Jianzhong Zhang, Bing Wu, Feng Peng, Libo Yuan

**Affiliations:** 1 Key Laboratory of In-Fiber Integrated Optics, Ministry of Education, Harbin Engineering University, Harbin 150001, China; lichuang@hrbeu.edu.cn (C.L.); yuzhangjun@hrbeu.edu.cn (Z.Y.); yuanyonggui@hrbeu.edu.cn (Y.Y.); zhangjianzhong@hrbeu.edu.cn (J.Z.); wubing@hrbeu.edu.cn (B.W.); pengfeng@hrbeu.edu.cn (F.P.); lbyuan@vip.sina.com (L.Y.); 2 College of Science, Harbin Engineering University, Harbin 150001, China; 3 College of Information and Communication Engineering, Harbin Engineering University, Harbin 150001, China

**Keywords:** optical fiber sensor, polarization-maintaining fiber, white light interferometer, polarization mode coupling

## Abstract

The high-order interference (HOI)—The interferogram introduced by polarization mode couplings (PMC) of multiple perturbations—Will cause misjudgment of the realistic coupling points in polarization-maintaining fiber (PMF) which is tested with a white light interferometer (WLI) with large dynamic range. We present an optical path tracking (OPT) method for simplifying the analysis of HOI, and demonstrate the enhancement and suppression conditions for the HOIs. A strategy is proposed to readily identify HOI by altering the spliced angle between polarizers’ pigtails and the PMF under test. Moreover, a PMF experiment with two perturbation points, for simplicity, is given as an example. As a result, all the characteristic interferograms including HOIs can be distinguished through just four measurements. Utilizing this identification method, we can estimate the realistic coupling points in PMFs and distinguish them from the interference signals including numerous HOIs.

## 1. Introduction

Polarization-maintaining fiber (PMF) is a crucial component of integrated optical sensors and fiber-optic interferometers [1,2]. Called polarization mode coupling (PMC), the optical power coupling between two orthogonal PMF polarization modes can be generated by inner structural imperfections or external perturbations along the PMF [3,4]. PMC could be utilized to evaluate the characteristics of polarization devices, such as the PER of Y-waveguides [5] and the angular alignment between PMFs [6]. Typical PMC measurements based on white light interferometer (WLI) focus on the 1st-order interference produced by the exciting mode and coupling mode with only one occurrence of PMC [7], because there is a consistent one-to-one correspondence between the 1st-order interference and the real perturbation point in the PMF under test. In reality, the light in the fast-axis caused from the coupling at a perturbation point will couple back to the slow-axis at the subsequent coupling points along fiber. If there are multiple perturbation points in a PMF under test, it will generate interference between the exciting mode and the coupling mode with more than one occurrence of PMC in the output signals of WLI. Additionally, the interference—We call it high-order interference (HOI)—Has been detected by employing the measurement system with a dynamic range of 90 dB reported in previous works [5,8].

As typical multiple coupling applications, the Lyot filter and Lyot depolarizer have been employed successfully in interferometric fiber optical gyroscopes (IFOGs) [9,10]. However, the HOI produced in PMF, which indicates no realistic coupling points, will be confused with the 1st-order interference without a clear analysis on the origin of HOI. For instance, the HOI in IFOG coil inspection and PMF-based sensors will cause misjudgment of the distribution of stress or spliced points. Litton Corporation has pointed out that there are thousands of possible interferences, including HOI, for a single-axis IFOG based on PMF and polarization-maintaining (PM) components [11]. In the case of distribution sensors, it has been reported that with merely several coupling points there will be many spurious interference signals. Chen *et al.* suggested that “spurious peaks” will occur inevitably due to many coupling points along PMF, and it has become one of the major problems that limits the multiplexing capacity of WLI systems [12]. Wang *et al.* analyzed the influence of the 2nd-order interference, which is called as the ghost coupling [13], on the distributed PMC measurements by using a rotatable half-wave plate [14]. Furthermore, these reports only focused on the 2nd-order interference and did not propose a universal method to identify HOI. Therefore, it is significant to determine the characteristics of HOI brought by multiple perturbation points in PMF. The traditional analysis methods for the polarization light transmission through polarization devices based on Jones matrix [15] or Mueller matrix [16] are also applicable to HOI. However, the computation will become extremely complex with the number increase of coupling points by these methods.

In this paper, an optical path tracking (OPT) method is presented for simplifying the analysis of polarization light transmission along PMFs with multiple perturbation points. A brief description of the OPT method is provided as the following three steps: (1) for a given scanning optical path difference (OPD), we divide an entire PMF into stable units in which the OPD is invariable; (2) we obtain the coupling intensity made by adjacent stable units and calculate the recursion formula; (3) we derive the general formulas of interference intensity for the entire PMF under test. It is demonstrated that the different HOIs will be suppressed or amplified depending on the different angle-related conditions. We present a method to identify HOI readily by altering the spliced angle between the polarizers’ pigtails and PMF under test with WLI system, which is verified by a simple case of two coupling points along a PMF experimentally. Finally, the system errors induced by the angle of polarizers and spliced points are discussed, and the variation trends of intensities are obtained for different HOIs.

## 2. Model and Analysis

### 2.1. WLI System with a Large Dynamic Range

**Figure 1:**
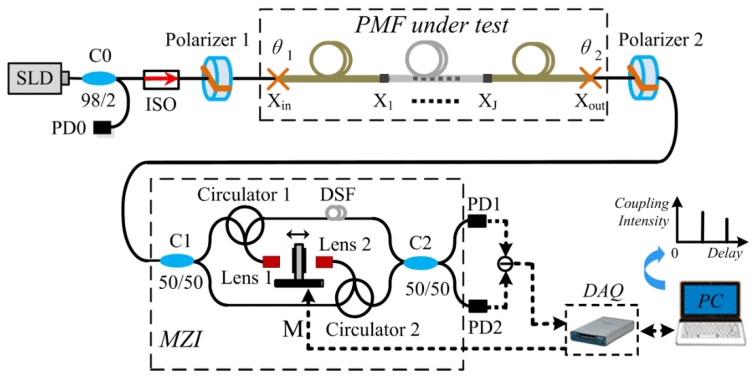
A distributed PMC measurement schematic for PMF. (C: coupler, PD: photodiode, ISO: Isolator, M: motor, MZI: Mach-Zehnder interferometer, DSF: dispersion-shifted fiber, DAQ: data acquisition) The PMF under test with multiple perturbation points (Points $X_{1}$, $X_{2}$, …, $X_{J}$) is spliced to Polarizers 1 and 2 at Points $X_{1}$ and $X_{2}$, respectively.

The PMC measurement setup for fiber sensors based on WLI is shown in Figure 1. The white light from a superluminescent light-emitting diode (SLD) is divided into two beams through a 98:2 fiber coupler. Two percent of the light is for monitoring the output power of the light source, and the remaining light is polarized by a 0°-rotated polarizer 1. Then the linearly polarized light is launched into the slow-axis of PMF under test. A part of linearly polarized light along the slow-axis will be coupled into the orthogonal axis at a perturbation point of PMF. Then it will generate two optical paths (OPs) with orthogonally eigenmodes, which will induce OPD due to the birefringence Δn of the PMF. Afterwards, the 1st-order coupling interferograms are detected with photodiodes (PD) by the scanning Mach-Zehnder interferometer (MZI) that will compensate the OPD.

The large dynamic range of system achieved in the previous works is improved from many aspects: Firstly, a differential detection is completed by adopting two PDs [8]. Secondly, the dispersion of fiber-based WLI is compensated by inserting a segment of dispersion-shifted fiber (DSF) into one arm of MZI. Thirdly, we utilize the differential scanning MZI with two lenses to suppress the optical power fluctuation [17]. As shown in Figure 1, a PMF with multiple perturbation points (denoted by point $X_{1}$, $X_{2}$, …, $X_{J}$) is tested by WLI. At each perturbation point, light is coupled not only from the polarization mode along the slow-axis to that along the fast-axis, but also from the polarization mode along the fast-axis to that along the slow-axis. As previously reported in [5], the resolution of the developed system can achieve nearly −90 dB, which can be utilized to evaluate the Y-waveguide with ultra-high PER. In this case, a great number of spurious interferograms, referring to the HOIs, will appear in spatial domain with large dynamic range.

### 2.2. Optical Path Tracking (OPT) Method

It has been recognized that a pair of OPs with an OPD less than the coherence length will suffer interference at the output of MZI and lead to an interferogram. For an identical scanning OPD in the spatial domain, there will be numerous possible pairs of OPs introduced by multiple perturbation points along PMF. The interferograms corresponding to the same scanning OPD with distinct OP pairs will give rise to the superposition of interference intensity. Therefore, the direct analysis of PMC for the entire PMF with multiple perturbation points, such as Jones matrix [15], will be rather complicated and cannot obtain the general formulas due to complex superposition phenomenon and the occurrence of HOIs.

Here, the OPT method based on the enumeration method and graphic method is presented to simplify the analysis of PMC. The steps of OPT method can be briefly described as follows: (1) Stable unit—we divide an entire PMF into stable units based on the corresponding OPD conditions and list all the OP pairs with graphic method; (2) Recursion formula—Then we obtain the recursion formula between arbitrary adjacent stable units; (3) General formulas—Finally we extend the recursion formulas to the entire PMF under test and derive the general formulas of interference intensity. With this method, the intensity and the order of interferograms could be identified for a given OPD.

#### 2.2.1. Stable Unit and Recursion Formula

We define the segment $\left(X_{j-p},X_{j}\right]$ (p≥1) of PMF as a stable unit with the following three characteristics: (a) the pair of OPs merely occurs once coupling between the orthogonal axes of PMF at the right end (Point $X_{j}$) of segment $\left(X_{j-p},X_{j}\right]$; (b) The position of $X_{j-p}$ satisfies that if we move it right until to Point $X_{j}$, the OPD of segment $\left(X_{j-p},X_{j}\right]$ is always invariable; (c) Point $X_{j-p}$ is chosen as the leftmost point that satisfies condition (b) in order to guarantee that all the stable units are linked end-to-end.

**Figure 2:**
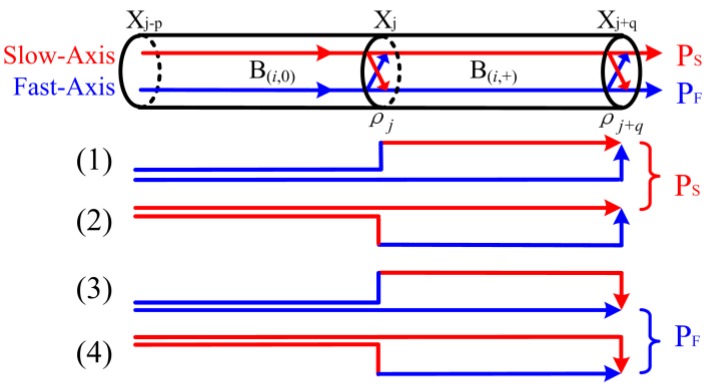
The graphics of any two adjacent units of PMF. Segment $\left(X_{j-p},X_{j+q}\right]$ are denoted by $B_{\left(i,0\right)}\cup B_{\left(i,+\right)}$ , where the subscript i represents the ith adjacent unit combination of PMF, the subscripts (0) and (+) represent the corresponding OPD = 0 and OPD ≠ 0, respectively, $X_{j-p}$, $X_{j}$ and $X_{j+q}\;\left(p,q\geq 1\right)$ are the perturbation points of PMF, respectively, $\rho_{j}$ is the coupling coefficient of the corresponding Point $X_{j}$, $P_{F}$ and $P_{S}$ are the light intensities out of the fast-axis and slow-axis of PMF, respectively.

Then stable unit can be classified into two categories based on the corresponding OPD introduced by the OP pairs in the segment, for simplicity, we denote stable unit by $B_{\left(i,0\right)}$ with OPD = 0 and $B_{\left(i,+\right)}$ with OPD ≠ 0, respectively. Obviously, the OPDs of arbitrary adjacent stable units are different, so that we might set the sequence of the *i*th adjacent units to $B_{\left(i,0\right)}\cup B_{\left(i,+\right)}$. As shown in Figure 2, the only four kinds of connections of adjacent units can be diagramed by enumeration method. The output intensity of the PMF Segment $\left(X_{j-p},X_{j+q}\right]$ from fast-axis and slow-axis at Point $X_{j+q}$ are denoted by $P_{X_{j+q},F}$ and $P_{X_{j+q},S}$, respectively, which can be evaluated as:

(1) $$\{\begin{array}{c} P_{X_{j+q},S}=P_{X_{j-p},S}(\rho_{j}\sqrt{1-\rho_{j}^{2}})(-\rho_{j+q}\sqrt{1-\rho_{j+q}^{2}})+P_{X_{j-p},F}(-\rho_{j}\sqrt{1-\rho_{j}^{2}})(-\rho_{j+q}\sqrt{1-\rho_{j+q}^{2}})\\ P_{X_{j+q},F}=P_{X_{j-p},F}(-\rho_{j}\sqrt{1-\rho_{j}^{2}})(\rho_{j+q}\sqrt{1-\rho_{j+q}^{2}})+P_{X_{j-p},S}(\rho_{j}\sqrt{1-\rho_{j}^{2}})(\rho_{j+q}\sqrt{1-\rho_{j+q}^{2}}) \end{array},p,q\geq 1$$

where, $\rho_{j}$ and $\rho_{j+q}$ are the coupling coefficients of the Point $X_{j}$ and $X_{j+q}$, respectively. The sign of $\rho_{j}$ changes only for coupling from the fast to the slow axis as shown in [18]. In most cases, it has the relation $\rho_{j}\ll 1$ in the detection for distributed polarization couplings along PMF [19]. Here, we are reasonable to neglect the slight errors introduced by the approximation $\sqrt{1-{\rho_{j}}^{2}}\approx 1$, which can be used to simplify the analysis. For any two adjacent units, Equation (1) can be rewritten as:

(2) $$\{\begin{array}{c} P_{i,S}=-(P_{i-1,S}-P_{i-1,F})\rho_{i,j}\rho_{i,j+q}\\ P_{i,F}=(P_{i-1,S}-P_{i-1,F})\rho_{i,j}\rho_{i,j+q} \end{array},i,q\geq 1$$

where, $P_{i,S}$ and $P_{i,F}$ represent light intensities from the slow-axis and fast-axis after passing through the ith adjacent units, respectively, $\rho_{i,j}$ and $\rho_{i,j+q}$ are the coupling coefficients of the point at the right end of Segment $\left(X_{j-p},X_{j}\right]$ and $\left(X_{j},X_{j+q}\right]$, respectively. From Equation (2), the stable units linked end-to-end can be expressed as:

(3) $$\{\begin{array}{c} P_{i,S}=-(P_{\text{In,S}}-P_{\text{In,F}})2^{i-1}\prod_{i=1}^{\max\{i\}}\rho_{i,j}\rho_{i,j+q}\\ P_{i,F}=(P_{\text{In,S}}-P_{\text{In,F}})2^{i-1}\prod_{i=1}^{\max\{i\}}\rho_{i,j}\rho_{i,j+q} \end{array},\;i,q\geq 1$$

where, $P_{\text{In,S}}$ and $P_{\text{In,F}}$ are the initial intensities that launch into the slow-axis and fast-axis of the first stable unit along PMF under test, respectively.

#### 2.2.2. Classifications and General Formulas

**Figure 3:**
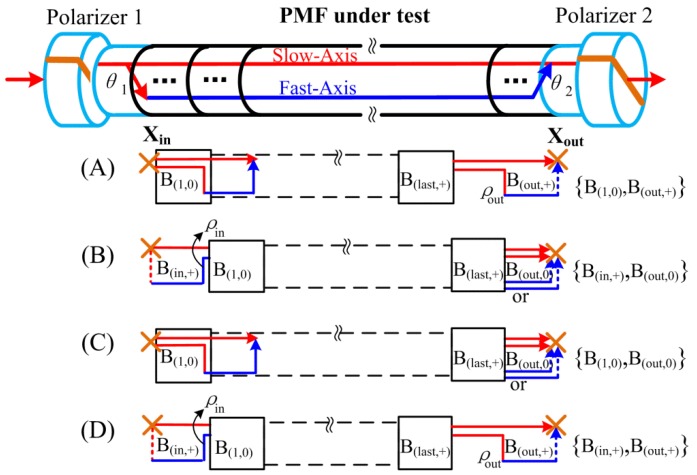
Depending on the two end unit types ($B_{\left(i,0\right)}$ or $B_{\left(i,+\right)}$), the scanning OPDs of the entire PMF under test are categorized into four kinds, which are notated by (A) {$B_{\left(1,0\right)}$, $B_{\left(\mathrm{out},+\right)}$}, (B) {$B_{\left(in,+\right)}$, $B_{\left(\mathrm{out},0\right)}$}, (C) {$B_{\left(1,0\right)}$, $B_{\left(\mathrm{out},0\right)}$} and (D) {$B_{\left(in,+\right)}$, $B_{\left(\mathrm{out},+\right)}$}, respectively. The consecutive units between the two black boxes in each kind conform with sequence of $B_{\left(i,0\right)}\cup B_{\left(i,+\right)}$. Besides, $\rho_{\text{in}}$ and $\rho_{\text{out}}$ represent the coupling coefficients of the points before the first unit $B_{\left(1,0\right)}$ and after the last unit $B_{\left(\mathrm{last},+\right)}$, respectively.

In this section, we consider that the pair of OPs of the first and last segments of the PMF under test. As mentioned above, we set the sequence of adjacent units as $B_{\left(i,0\right)}\cup B_{\left(i,+\right)}$ to simplify the analysis. However, the two end segments of the entire PMF under test might not be always satisfied the sequence. The OPD of the first and last segments could also conform to the sequence of $\left\{B_{\left(\text{in},+\right)}\cup (B_{\left(1,0\right)}\cup B_{\left(1,+\right)})\cup \cdot \cdot \cdot \right\}$ and $\left\{\cdot \cdot \cdot \cup (B_{\left(\mathrm{last},+\right)}\cup B_{\left(\mathrm{last},+\right)})\cup B_{\left(\mathrm{out},0\right)}\right\}$, respectively, where the first segment $B_{\left(\text{in},+\right)}$ and the last segment $B_{\left(\text{out},0\right)}$ satisfy the features of $B_{\left(i,+\right)}$ and $B_{\left(i,0\right)}$, respectively. Therefore, the scanning OPDs of the entire PMF can be categorized into four classifications based on the possible end segments conditions. As shown in Figure 3, the scanning OPDs of the entire PMF, for simplicity, are denoted by (A) {$B_{\left(1,0\right)}$, $B_{\left(\mathrm{out},+\right)}$}, (B) {$B_{\left(\mathrm{in},+\right)}$, $B_{\left(\mathrm{out},0\right)}$}, (C) {$B_{\left(1,0\right)}$, $B_{\left(\mathrm{out},0\right)}$} and (D) {$B_{\left(\mathrm{in},+\right)}$, $B_{\left(\mathrm{out},+\right)}$}, respectively.

The initial intensities ($P_{\text{In,S}}$ and $P_{\text{In,F}}$) and terminal intensities ($P_{\text{Out,S}}$ and $P_{\text{Out,F}}$) for the four conditions in Figure 3 are expressed as:

(4a) $$\{\begin{array}{cc} P_{\text{In,S}}=\cos^{2}\theta_{1}\;,\;P_{\text{In,F}}=\sin^{2}\theta_{1}, & \text{first segment}\in B_{\left(i,0\right)}\\ P_{\text{In,S}}=\sin\theta_{1}\cos\theta_{1}(-\rho_{\mathrm{in}}),P_{\text{In,F}}=\sin\theta_{1}\cos\theta_{1}\rho_{\mathrm{in}}, & \text{first segment}\in B_{\left(i,+\right)} \end{array}$$

(4b) $$\{\begin{array}{cc} P_{\text{Out,S}}=P_{i,S}\cdot \cos^{2}\theta_{2}\;,\;P_{\text{Out,F}}=P_{i,F}\cdot \sin^{2}\theta_{2}, & \text{last segment}\in B_{\left(i,0\right)}\\ P_{\text{Out,S}}=P_{i,S}\cdot \rho_{\text{out}}(-\sin\theta_{2})\cos\theta_{2},P_{\text{Out,F}}=P_{i,F}\cdot (-\rho_{\text{out}})(-\sin\theta_{2})\cos\theta_{2}, & \text{last segment}\in B_{\left(i,+\right)} \end{array}$$

where, $\rho_{\text{in}}$ and $\rho_{\text{out}}$ are the coupling coefficients of the points before the first unit $B_{\left(1,0\right)}$ and after the last unit $B_{\left(\mathrm{last},+\right)}$, respectively, $P_{\text{Out,S}}$ and $P_{\text{Out,F}}$ represent the output intensity from slow-axis and fast-axis at spliced point $X_{\text{out}}$, respectively. Because the polarizer is aligned to the slow-axis of its PM pigtail, the amplitude changing of polarized light that launched into slow-axis of PMF at point $X_{\text{in}}$ is $\cos\theta_{1}$, and that coupled into fast-axis is $\sin\theta_{1}$. It is similar at the spliced point $X_{\text{out}}$. Therefore, the final interference intensity with a given OPD based on Equations (3) and (4) can be expressed as:

(5) $$\begin{array}{c} \left|P\right|=\left|P_{\text{Out,S}}+P_{\text{Out,F}}\right|\\ \;=\{\begin{array}{cc} 2^{i-1}T_{i}\rho_{\text{out}}\cdot \cos2\theta_{1}\sin2\theta_{2}, & \text{OPD}\in \{B_{\left(1,0\right)},B_{\left(\mathrm{out},+\right)}\}\\ 2^{i-1}T_{i}\rho_{\text{in}}\cdot \sin2\theta_{1}\cos2\theta_{2}, & \text{OPD}\in \{B_{\left(\mathrm{in},+\right)},B_{\left(\mathrm{out},0\right)}\}\\ 2^{i-1}T_{i}\cdot \cos2\theta_{1}\cos2\theta_{2}, & \text{OPD}\in \{B_{\left(1,0\right)},B_{\left(\mathrm{out},0\right)}\}\\ 2^{i-2}T_{i}\rho_{\text{in}}\rho_{\text{out}}\cdot \sin2\theta_{1}\sin2\theta_{2}, & \text{OPD}\in \{B_{\left(\mathrm{in},+\right)},B_{\left(\mathrm{out},+\right)}\} \end{array},\;T_{i}=\{\begin{array}{cc} \prod_{i=1}^{\max\{i\}}(\rho_{i,j}\rho_{i,j+q}), & i\geq 1\\ 1, & i=0 \end{array} \end{array}$$

where, i=0 represents there is no stable unit $B_{\left(i\right)}$. In addition, the central interferogram intensity is calculated as $\left|P_{central}\right|=\cos^{2}\theta_{1}\cos^{2}\theta_{2}+\sin^{2}\theta_{1}\sin^{2}\theta_{2}$.

In reality, it might occur negative stable unit denoted by $B_{\left(i,-\right)}$ while there exist a positive term $B_{\left(i,+\right)}$. Here, the connection conditions of adjacent units can be classified to (a) $B_{\left(i,+\right)}\cup B_{\left(i,0\right)}\cup B_{\left(i,-\right)}$ and (b) $B_{\left(i,0\right)}\cup B_{\left(i,+\right)}\cup B_{\left(i,-\right)}$. Similar to the above analysis, we generalize the results as follows. In case of (a), the interference intensities with given OPD situations are unchanged. In case of (b), the interference intensities only should be multiplied by $\rho_{i}^{2}$ instead of the corresponding $\rho_{i}$, which is produced at the corresponding kink point between $B_{\left(i,+\right)}$ and $B_{\left(i,-\right)}$, and the other terms are remained the same.

Some summaries can be acquired by the above analysis, if we define the interference-order as $N=N_{1}+N_{2}+\cdot \cdot \cdot +N_{i}$ that can be found in the coupling coefficients term $\rho_{1}^{N_{1}}\cdot \rho_{2}^{N_{2}}\cdot \cdot \cdot \rho_{i}^{N_{i}}(N_{i}=0,1,2)$ in Equation (5). Because there are obviously even-number times couplings in arbitrary adjacent two units, the interference-order *N* of the four conditions in Figure 3 can be summarized as N∈ odd-order when OPD∈ case (A) or (B), and N∈ even-order when OPD∈ case (C) or (D). Note that the intensities of every interferogram are related to the inject angle $\theta_{1}$ at polarizer 1 and the output angle $\theta_{2}$ at polarizer 2 in Equation (5). Especially, 45° and 0° for $\theta_{1}$ or $\theta_{2}$ would introduce interesting results. The intensity of odd-order interferences have the maximum and even-order interferences are reduced to zero when $\theta_{1}$ − $\theta_{2}$ are 0°–45°, or 45°–0°, respectively. However, the variation trend of intensities are the exactly opposite results when $\theta_{1}$ − $\theta_{2}$ are 0°–0°, or 45°–45°, respectively. Therefore, we can identify HOI by altering the spliced angle between polarizers’ pigtails and the PMF.

## 3. Experimental Results

### 3.1. Theoretical Estimation

A PMF (segment $X_{I}X_{O}$) including two perturbation points $X_{A}$ and $X_{B}$, for simplicity, is demonstrated experimentally to prove the model of HOI introduced by PMC. The OPD denoted by $S_{MN}$ (MN = IA, AB, and BO) between two adjacent points M and N (segment MN) can be calculated as:

(6) $$S_{MN}=\Delta n\cdot l_{MN}$$

where, $l_{MN}$ represents the length of corresponding PMF section ($l_{\text{IA}}$ = 2.16 m, $l_{\text{AB}}$ = 5.22 m, and $l_{\text{BO}}$ = 16.56 m), and the birefringence Δn of this PMF is about 5.6 × 10^−4^.

Then, the $S_{IO}$, refers to the OPD of the whole PMF, can be expressed as $S_{IO}=\sum \alpha \cdot S_{MN}\;(\alpha =0,\pm 1)$. All the different kinds of $S_{IO}$ are enumerated readily utilizing emulation tool. It seems obvious that there will be $(\beta^{3}-1)/2$ kinds of $S_{IO}$ when we only consider the positive values of $S_{IO}$, where β is the number of segment MN along PMF. Finally, we choose the corresponding formula (see Equation (5)) based on the different OPD of the first and last segments to acquire the interference intensity. Besides, for a given $S_{IO}$, the interference-order *N* will be determined by the unique formula. In the case of two perturbation points, there will be 13 possible interferograms with different scanning OPD (the positions and interferogram coefficients are listed in Table 1).

### 3.2. Identification of HOI and Results

**Figure 4:**
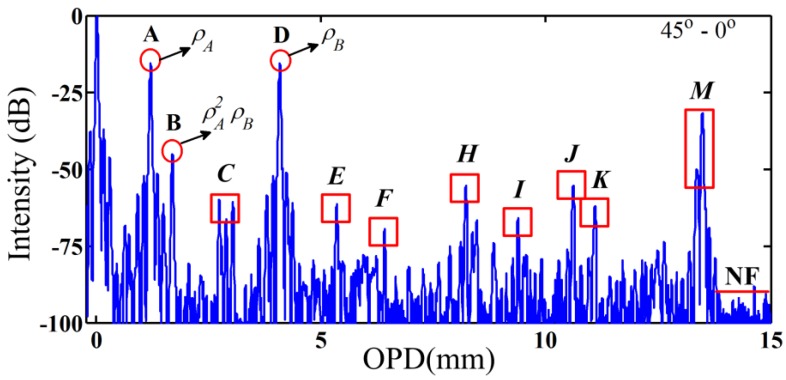
Experimental results of a PMF with the angle combination of 45°–0°. Interferograms A, B, and D can be directly identified by Equation (5). The notation NF represents the noise floor of the interference signal, which indicates the sensitivity of measurement system.

It has been recognized that we could set the angle of input-output angles of polarizers of WLI to 45°–0° or 0°–45° for testing the PMF sensors or IFOG coils. In these cases, even-order interferences are suppressed and only the 1st-order and 3rd-order interferences are exposed. The envelopes of interferograms *versus* scanning OPD with the angle combination 45°–0° are plot in Figure 4. Three interferograms could be forecast as expressed in Equation (5). The 1st-order interference denoted by interferograms A and D correspond to points $X_{A}$ and $X_{B}$, respectively, and interferogram B represents the 3rd-order interference whose intensity is $\rho_{A}^{2}\rho_{B}$.

**Figure 5:**
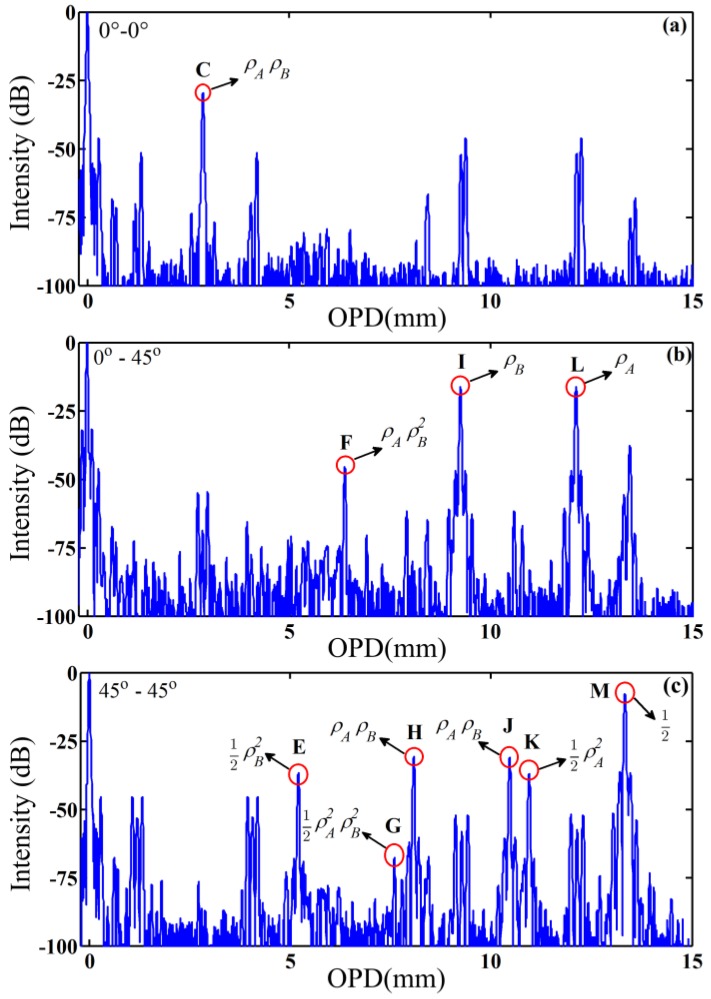
Experiment results of a PMF with additional three angle combinations of (**a**) 0°–0°; (**b**) 0°–45° and (**c**) 45°–45°. They demonstrate the enhancement or suppression of the interferograms marked with box in Figure 4, which can be used to identify the HOI.

However, there are numerous extra interferograms without explicit meanings, which are marked by the red boxes. We only need to determine whether the interferogram intensities could be amplified by altering the spliced angle between the pigtails of polarizers and PMF according to Equation (5). Subsequently, the spliced angle combinations are set to 0°–45°, 0°–0°, and 45°–45°, respectively, and attention is paid to the intensity variation at the corresponding positions of interferograms *C*, *E*, *F*, *H*, *I*, *J*, *K*, and *M* in Figure 4. As shown in Figure 5a,c, the intensities of interferograms *C*, *E*, *H*, *J*, and *K*, which represent the 2nd-order interference, and that of interferogram *M*, which represents the 0th-order interference, are enhanced to their maximum. Figure 5b shows that the intensity of interferogram *F*, which represents the 3rd-order interference, and that of interferogram *I*, which represents the 1st-order interference, are increased to the maximum. In consequence, all the interferograms marked by red boxes in Figure 4 can be identified through just four times of measurement at different angles between the pigtails of polarizers and PMF under test. We can extract realistic signals (1st-order interference) and eliminate spurious signals from the results to evaluate the polarization characteristics of PMF.

It shall be noticed that there are several unexpected interferograms around interferograms, such as interferograms *C* or *M*. The reason lies in that the short PM pigtails of the two added polarizers are not taken into consideration for the proposed model. In these experiments, the lengths of PMF polarizers’ pigtails are 0.30 m and 0.25 m, respectively. The pigtails could be considered and analyzed as another two segments of PMF, which will lead to the side interferograms around the characteristic interferograms.

## 4. Discussions

The positions and intensities of the total interferograms shown in Figure 4 and Figure 5 are listed in Table 1. The 1st-order coupling corresponding to Points $X_{A}$ and $X_{B}$ are 14.9 dB and 15.0 dB, respectively, which are measured by a PER meter (ERM-102, General Photonics, Chino, CA, USA). The 2nd to 4th-order interferences could be calculated based on the 1st-order coupling. The errors are less than 2 dB, which might be caused by the small misalignments (<1°) induced by fiber fusion splicer and PMF dispersion.

**Figure 6:**
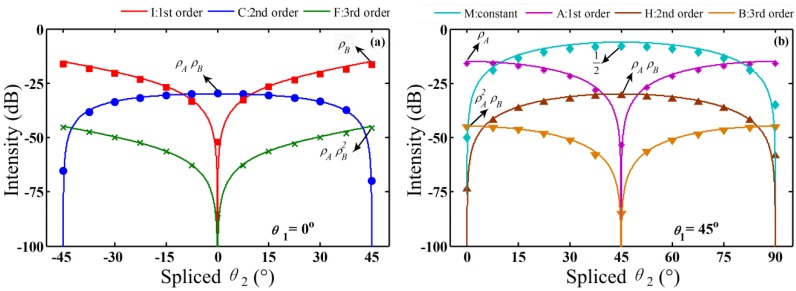
The intensity variation trend of different orders’ HOIs with varying angle $\theta_{2}$. The input polarizer 1 angle $\theta_{1}$ is set to 0° (**a**) and 45° (**b**), respectively. The experimental results are marked by different dot notations, and the theoretical curves are expressed by the solid lines. The maximum values of each curve represent the meaning of coupling intensities at the corresponding scanning OPD.

In order to further verify the HOI variation trend obtained by OPT method, the intensity variations of some interferograms including interference signals ranging from 0th-order to 3rd-order are measured. With the input polarized angle $\theta_{1}$ fixed at 0° and 45°, the output angle $\theta_{2}$ are change by 7.5° step by step, respectively. It can be seen from Figure 6a, when $\theta_{1}$ is set at 0°, interferogram *C* decreases to −70 dB with $\theta_{2}$ = 45°, and interferograms *I* and *F* reduce to less than –50 dB and −90 dB with $\theta_{2}$ = 0°, respectively. Similarly in Figure 6b, when $\theta_{1}$ is set at 45°, interferogram A of the 1st-order interference and interferogram B of the 3rd-order interference are suppressed with $\theta_{2}$ = 45°, and interferogram *H* of the 2nd-order interference and interferogram *M* of the 0th-order interference are suppressed with $\theta_{2}$ = 0°. Because of the spliced angle errors and the manufacture errors of added polarizers, the HOI and the 1st-order interference interferograms cannot be eliminated completely at the maximum slope angles as shown in Figure 6. Therefore, these HOIs can be identified far away from the maximum slope angle combinations.

The proposed method is helpful to realize the complicated OPs behaviors transmitted along PMF with perturbation points. Based on the results and discussions, the realistic coupling introduced by the splice points of PMF could be identified readily from the interference signals. As shown in Figure 4, interferograms A and D correspond to the real perturbation points X_A_ and X_B_, respectively. Meanwhile, interferograms I and L also represent the points X_B_ and X_A_, respectively, due the opposite spliced angle combinations (see Figure 5b). Besides, we can choose the angle combination conditions to control the occurrence of HOIs to acquire appropriate presentation. For the devices based single-variety such as the IFOG coil, we could directly set the angle of input-output polarizers of PMC measurement system to 0°–45° or 45°–0°. In this case, 2nd-order interference is suppressed and 1st-order interference is shown out to evaluate devices performance. For the high-precision integrated devices such as the IFOG system which contain the connection or splice points between different components, we could adopt the angle combinations of input-output polarizers of 0°–0° or 45°–45° to suppress the weaker 1st-order interference.

## 5. Conclusions

The HOI introduced by the PMCs of multiple perturbation points in PMFs is analyzed in detail. An OPT method—Based on the enumeration method and graphic method—Is presented for simplifying the analysis of polarization light transmission along PMF with multiple perturbation points. The positions and intensities of HOI interferograms can be calculated by the derived general HOI formulas utilizing OPT method. It is demonstrated that the odd-order or even-order HOIs will be suppressed or amplified depending on the angle between the added pigtails of polarizers and the PMF under test. Furthermore, the method is verified by a case of two coupling points along a PMF by a WLI system. As a result, all the characteristic interferograms including HOIs can be distinguished through just four measurements. The identification method is useful to evaluate the polarization performance of PMF, suppress the system noise of WLI and improve its sensitivity.

## Figures and Tables

**Table 1:** Interferogram measurement results.

Interferogram	Position Meaning	Interferogram Meaning	Position (mm)	Normalized Intensity/Error (dB)				Order *N*
				0°–0°	0°–45°	45°–0°	45°–45°	
	0	1	0	0	0	0	0	
**M**	$\left|S_{IA}+S_{AB}+S_{BO}\right|$	12	13.33	<−70	<−50	<−50	−7.8/1.8	0th
**A**	$\left|S_{IA}\right|$	$\rho_{A}$	1.22	<−70	<−70	−15.6/0.7	<−50	1st
**D**	$\left|S_{IA}+S_{AB}\right|$	$\rho_{B}$	4.09	<−70	<−70	−15.7/0.7	<−50	
**I**	$\left|S_{BO}\right|$	$\rho_{B}$	9.27	<−50	−16.5/1.5	<−70	<−50	
**L**	$\left|S_{AB}+S_{BO}\right|$	$\rho_{A}$	12.12	<−50	−16.3/1.4	<−70	<−50	
**C**	$\left|S_{AB}\right|$	$\rho_{A}\rho_{B}$	2.87	−29.7/0.2	<−60	<−60	<−70	2nd
**E**	$\left|S_{IA}+S_{AB}-S_{BO}\right|$	12ρB2	5.21	<−70	<−70	<−70	−36.7/0.7	
**H**	$\left|S_{IA}-S_{BO}\right|$	$\rho_{A}\rho_{B}$	8.08	<−70	<−70	<−70	−30.6/0.7	
**J**	$\left|S_{IA}+S_{BO}\right|$	$\rho_{A}\rho_{B}$	10.47	<−70	<−70	<−70	−31.0/1.1	
**K**	$\left|S_{IA}-S_{AB}-S_{BO}\right|$	12ρA2	10.95	<−70	<−70	<−70	−36.9/1.1	
**B**	$\left|S_{IA}-S_{AB}\right|$	$\rho_{A}^{2}\rho_{B}$	1.70	<−70	<−70	−45.1/0.3	<−70	3rd
**F**	$\left|S_{AB}-S_{BO}\right|$	$\rho_{A}\rho_{B}^{2}$	6.38	<−70	−45.7/0.8	<−60	<−70	
**G**	$\left|S_{IA}-S_{AB}+S_{BO}\right|$	12ρA2ρB2	7.60	<−70	<−70	<−70	−67.7/1.9	4th
